# Understanding the wider needs of children with disabilities

**Published:** 2013

**Authors:** Maria Zuurmond

**Affiliations:** Research fellow: International Centre for Evidence in Disability, London School of Hygiene and Tropical Medicine, London, UK.

**Figure:**
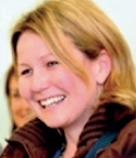
Maria Zuurmond

It is easy for all of us to focus in on our own area of expertise, and treat just one aspect of a child's health. For the typical community eye worker this focus is, of course, on providing eye care. In reality, most children and their families will have much broader needs and priorities. Without addressing these other issues, or at least referring families to other services, your work may have less impact.

In this article we share some experiences from a parent training project in Bangladesh, funded by CBM and carried out in partnership with the Child Sight Foundation, which aims to better equip parents to provide care for children with cerebral palsy. Many children with this condition will have a range of other health issues, which can include being visually impaired.

A key first step in our project was to undertake initial interviews with families in order to better understand their priorities.

We also wanted to understand, by talking to the children, what their daily lives were like and what was important to them. Being able to go to school was a priority for some children, was the importance of not only focussing our support on access to health services, but also on improved access to education.

**Figure:**
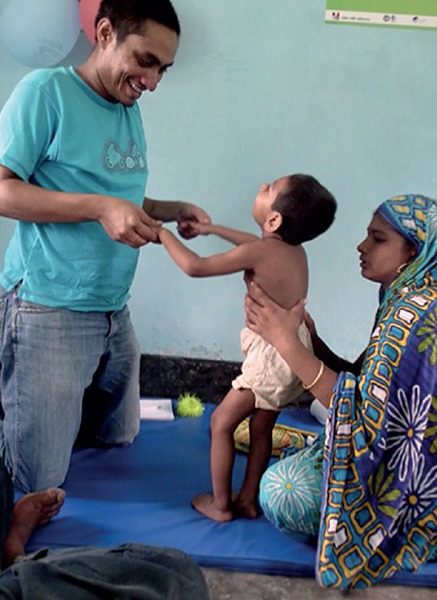
Training for parents of children with cerebral palsy. BANGLADESH

## What can eye care workers do?

As an eye care worker you cannot do everything. But the examples below illustrate that it is really important to take some time to find out what other services exist in your local community, and how best you can refer families to these services. In Sonia's case, we found out what nutrition programmes there were in the district and made contact with them.

It is a good idea to think outside the health or eye care ‘box’. It might be that you can link a family to a non-governmental organisation working on livelihoods and income-generating activities. Or you might find time to promote your services to a local school and talk to the teachers about how they can be more inclusive of children with visual impairments. By reaching out and connecting children with services, you can make a difference in the quality of their lives.

**Case studies**

**Sonia's story**

Sonia* lived with her parents and two younger siblings in a rural district in Bangladesh. She had multiple disabilities, was unable to stand unaided, and had both visual and hearing impairments. When we met her mother for the interview, it was obvious that Sonia was malnourished, and that she had a skin condition and other health problems. She spent most of her time sitting on a red plastic chair outside of the house; she didn't play with anything because her mother explained that she was unable to hold anything in her hand.

Sonia was diagnosed with cerebral palsy during an earlier medical camp, and was referred and treated for two cataract operations, which were both provided free of charge. She was also given glasses, but never wore them, and was beginning to develop posterior capsule opacification.

Her mother explained that one of the family's main challenges was the fact that Sonia was often ill, for example with pneumonia, and they had already sold three cows to pay for her treatment. Feeding and toileting were identified as their main concerns in caring for Sonia: “The hardest thing is the feeding, and more than that is the toileting… Before she was able to eat more food than now. She used to take some bread and milk, but now the only thing she takes is some rice and milk. The rice has to be mashed up. She will only take 3–4 tablespoons and then refuses it. This has been for the last 3–4 months.”

This interview raised important issues for us in planning our own parent training project. We were completely focused on providing a training course, but clearly there were a range of broader health issues that also needed to be addressed. We couldn't conduct the training in isolation. We needed to explore links which could be forged with local nutrition programmes, and also how to improve access to address primary health care needs.

Very sadly, since conducting this interview, Sonia has died.

**Atia's story**

Atia* is 14 years old and lives with her two younger sisters and her parents. She has cerebral palsy, cannot walk, and is completely dependent upon members of her family for all her personal care. She loves to attend school, although she cannot always get to school because it is not an easy journey. Perhaps this is one of the reasons why she has been studying in the same class (Grade 1) for the last three years, and the rest of the children in the class are younger than her. Her mother takes her to school in a wheelchair, but when crossing from the main road to the side road her mother has to carry her, and her younger sister has to carry the wheelchair. This is not an easy task, especially as Atia gets older.

**‘I like it when my friends come to me at the school’**

Atia explains to us what make her feel both happy and sad: “If my mother does not take me to the school I feel sad. My mother is busy, which is why sometimes she can't take me to school. Taking me to the school is very pain-tasking job for my mother; I feel bad seeing her pain.”

“I like it when my friends come to me at the school. They help me with crossing the stairs and they don't get angry. Teachers are good at school. They teach us, they don't get angry. They love me. They call me by my name and ask me how I am doing – that's why I feel good.”

*All names in this article have been changed.

